# Association between Human Blood Proteome and the Risk of Myocardial Infarction

**DOI:** 10.31083/j.rcm2506199

**Published:** 2024-05-30

**Authors:** Linghuan Wang, Weiwei Zhang, Zhiyi Fang, Tingting Lu, Zhenghui Gu, Ting Sun, Dong Han, Yabin Wang, Feng Cao

**Affiliations:** ^1^Department of Medicine School, Nankai University, 300071 Tianjin, China; ^2^Department of Cardiology, National Research Centre for Geriatric Diseases & National Key Lab for Chronic Kidney Disease & Second Medical Centre of Chinese PLA General Hospital, 100853 Beijing, China; ^3^Chinese PLA Medical College & Department of Cardiology, National Clinic Research Center Geriatric Disease, 2nd Medical Center of Chinese PLA General Hospital, 100853 Beijing, China

**Keywords:** myocardial infarction, human blood proteome, killer cell immunoglobulin-like receptor 2ds2, vacuolar protein sorting-associated protein 29, cardiotrophin-1, Selenoprotein S, histo-blood group ABO system transferase

## Abstract

**Background:**

The objective of this study is to estimate the causal relationship between plasma proteins and myocardial infarction (MI) through Mendelian randomization (MR), predict potential target-mediated side effects associated with protein interventions, and ensure a comprehensive assessment of clinical safety.

**Methods:**

From 3 proteome genome-wide association studies (GWASs) involving 9775 European participants, 331 unique blood proteins were screened and chosed. The summary data related to MI were derived from a GWAS meta-analysis, incorporating approximately 61,000 cases and 577,000 controls. The assessment of associations between blood proteins and MI was conducted through MR analyses. A phenome-wide MR (Phe-MR) analysis was subsequently employed to determine the potential on-target side effects of protein interventions.

**Results:**

Causal mediators for MI were identified, encompassing cardiotrophin-1 (CT-1) (odds ratio [OR] per SD increase: 1.16; 95% confidence interval [CI]: 1.13–1.18; *p* = 1.29 × ${\mathrm{10}}^{-31}$), Selenoprotein S (SELENOS) (OR: 1.16; 95% CI: 1.13–1.20; *p* = 4.73 × ${\mathrm{10}}^{-24}$), killer cell immunoglobulin-like receptor 2DS2 (KIR2DS2) (OR: 0.93; 95% CI: 0.90–0.96; *p* = 1.08 × ${\mathrm{10}}^{-5}$), vacuolar protein sorting-associated protein 29 (VPS29) (OR: 0.92; 95% CI: 0.90–0.94; *p* = 8.05 × ${\mathrm{10}}^{-13}$), and histo-blood group ABO system transferase (NAGAT) (OR: 1.05; 95% CI: 1.03–1.07; *p* = 1.41 × ${\mathrm{10}}^{-5}$). In the Phe-MR analysis, memory loss risk was mediated by CT-1, VPS29 exhibited favorable effects on the risk of 5 diseases, and KIR2DS2 showed no predicted detrimental side effects.

**Conclusions:**

Elevated genetic predictions of KIR2DS2 and VPS29 appear to be linked to a reduced risk of MI, whereas an increased risk is associated with CT-1, SELENOS, and NAGAT. The characterization of side effect profiles aids in the prioritization of drug targets. Notably, KIR2DS2 emerges as a potentially promising target for preventing and treating MI, devoid of predicted detrimental side effects.

## 1. Introduction

In Western countries, myocardial infarction (MI) and coronary artery disease are the primary pathologies for increased mortality [1], and are an increasing burden on global health even with highly effective statin therapy [2]. Coronary heart disease may initially be assymptomatic, and often presents as a major adverse event, such as a MI [3]. Therefore, new and improved strategies for the treatment and prevention of MI are needed. Plasma proteins are pivotal in the biological processes of a range of diseases [4, 5] and serve as the primary therapeutic targets for treatment and prevention [6, 7, 8]. Plasma proteins, due to their physical interaction with blood vessels, play a crucial role in circulatory disease pathophysiology.

Mendelian randomization (MR) employs genetic variants as instrumental variables to examine the causal impact of risk factors on outcomes. Due to the fixed nature of genetic variants at conception, the method is immune to biases arising from reverse causality [9, 10]. The results of MR are very similar to those of randomized controlled trials, and as a result, MR has gained increasing popularity as a method to provide more robust estimates for the causal effects of various risk factors on a spectrum of diseases [11, 12, 13]. Identifying genetic variants associated with proteins through genome-wide association studies (GWAS) of plasma protein levels [13, 14, 15, 16] allows assessing the causal impact of potential drug targets through MR [16, 17].

Conducting a systematic MR, we sought to estimate the causal effects of plasma proteins on MI. We initially a systematic MR study involving 331 plasma proteins to pinpoint potential mediators of MI. Subsequently, a phenome-wide MR (Phe-MR) analysis was utilized to reveal unexpected adverse effects and explore possibilities for drug therapy. This analysis is designed to predict potential target-mediated side effects linked to protein interventions, ensuring a more thorough evaluation of clinical safety.

## 2.Methods

### 2.1 Study Design

**Fig. 1.**
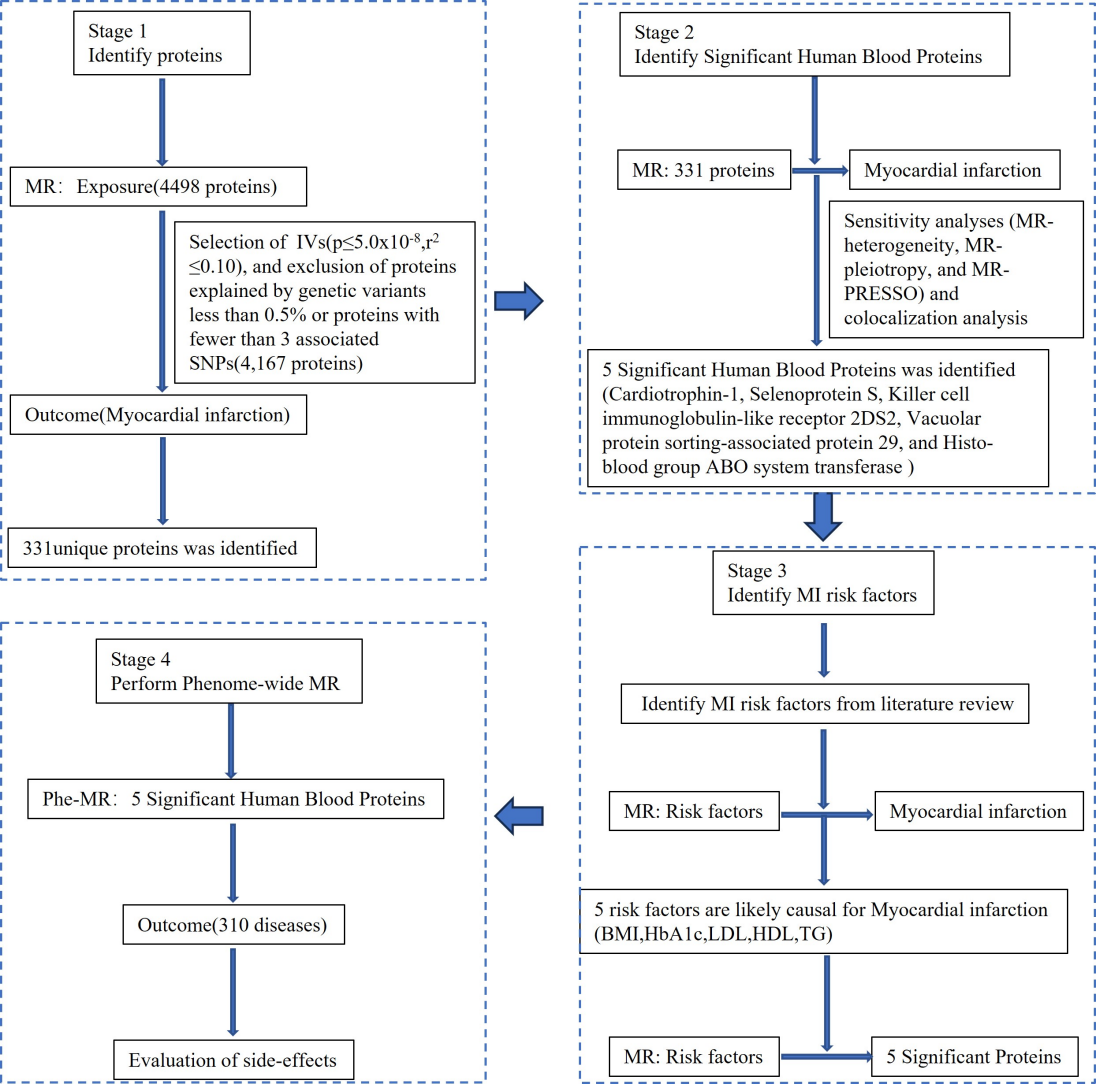
**Conceptual framework of Mendelian randomization (MR) study. **IVs, instrumental variable; SNPs, single nucleotide polymorphisms; MI, myocardial infarction; PRESSO, Pleiotropy RESidual Sum and Outlier; ABO, Blood types A, B, and O Blood Typing System; BMI, body mass index; HbA1c, glycosylated hemoglobin, type A1C; LDL, low-density lipoprotein; HDL, high-density lipoprotein; TG, triglyceride; Phe-MR, phenome-wide MR.

Following the STrengthening the Reporting of OBservational studies in Epidemiology (STROBE)-MR guideline, the current study ensured adherence to the standards for reporting observational studies in epidemiology using MR [18]. Conducting a four-stage MR study, potential drug targets for MI were systematically identified, as illustrated in Fig. 1. MR design relies on 3 core assumptions: (1) the direct impact of genetic variants on exposures; (2) the lack of association between genetic variants and potential confounders; and (3) the influence of genetic variants on outcomes occurs solely through their effects on exposures [19]. Utilized in the present study were the summary-level data from publicly available European-descent GWASs for the blood proteome, MI, and 310 non-MI diseases. Approval for the protocol and data collection was granted by the ethics committee of the original GWASs. Written informed consents were obtained prior to the commencement of data collection.

### 2.2 Data

This study enrolled a total of 9775 European individuals from 3 large-scale GWASs from which the summary data of single nucleotide polymorphisms (SNPs) associated with the human proteome, serving as genetic instruments, was obtained (Table 1, Ref. [13, 20, 21]). The analysis conducted by Sun *et al*. [13] involved 3282 proteins in 3301 participants, utilizing 10,534,735 SNPs from the INTERVAL study. Folkersen *et al*. [20] analyzed 83 proteins in 3394 participants with 5,270,646 SNPs from the IMPROVE study. Suhre *et al*. [21] analyzed 1124 proteins in 3080 participants with 501,428 SNPs from the KORA F4 study and the QMDiab assay (Table 1). The Integrative Epidemiology Unit (IEU) GWAS database provided the public databases for the aforementioned proteins (https://gwas.mrcieu.ac.uk/).

**Table 1.** **Characteristics of human blood proteome GWASs used for genetic instruments selection**.

Reference	Cohort (s)	Description	Sample size	Country	Population	Number of human blood proteins analyzed	Number of SNPs
Sun *et al*. [13]	INTERVAL study	randomized trial	3301	England	European	3282	10,534,735
Folkersen *et al*. [20]	IMPROVE study	multicenter, observational study	3394	Finland, France, Italy, the Netherlands, and Sweden	European	83	5,270,646
Suhre *et al*. [21]	KORA F4 study	population-based cohort	3080	German	European	1124	501,428
	QMDiab	cross-sectional case-control study		Qatar			
Total			9775			4498	16,306,809

GWASs, genome-wide association studies; SNPs, single nucleotide polymorphisms; QMDiab, Qatar Metabolomics Study on Diabetes.

The summary genetic statistics for MI were obtained from the Coronary ARtery DIsease Genome wide Replication And Meta-analysis (CARDIoGRAM) plus C4D investigators and The UK BioBank [22] (**Supplementary Table 1**). The UK Biobank included 17,505 cases and 454,212 controls with a total of 10,903,881 SNPs. CARDIoGRAM plus C4D included ~44,000 MI cases and ~123,504 controls with a total of 9,289,491 SNPs. Finally, ~61,000 MI cases and ~577,000 controls with 8,126,035 SNPs common to both data sets were obtained [22]. The diagnosis for MI was made by fulfilling any one of the following criteria [23]: (1) An increase and/or decrease in cardiac biomarkers with at least one value surpassing the 99th percentile of the upper reference limit, coupled with evidence of myocardial ischemia. (2) Sudden, unexpected cardiac death, often accompanied by symptoms suggestive of MI and electrocardiogram (ECG) changes indicative of new ischemia. (3) Conditions consistent with perioperative myocardial necrosis and percutaneous coronary intervention (PCI)-related/coronary artery bypass grafting (CABG)-related MI. (4) Pathological observations suggestive of an acute MI.

### 2.3 Genetic Instruments for Blood Proteins

Using MR, we utilized SNPs at a genome significance level of *p* value < 5 × ${\mathrm{10}}^{-8}$. These SNPs were independent of other SNPs ($r^{2}$ < 0.1) and served as instruments for these proteins. The plasma cis-protein quantitative trait loci (pQTLs) were considered as instruments. In cases where protein-associated SNPs were not present in the MI dataset, a proxy SNP ($r^{2}$ > 0.8) was automatically chosen for the MR analysis. Following this, we computed the phenotypic variance explained by each blood protein’s corresponding instruments. To ensure sufficient statistical power, proteins with less than 0.5% variance explained by genetic variants were excluded [24]. Furthermore, we excluded proteins associated with fewer than 3 SNPs since certain MR sensitivity analyses necessitate a minimum of 3 SNPs associated with the exposure [25, 26].

Finally, the MR analysis included a total of 331 unique blood proteins, with 4167 out of the initial 4498 proteins being excluded (Fig. 1). The strength of the genetic instruments for blood proteins was assessed using the F statistic, with a higher F-statistic (F >10) indicating a stronger instrument [27].

### 2.4 Statistics

In the primary analysis, we employed the inverse-variance weighted (IVW) MR method to assess the associations between 331 blood proteins and MI [28]. To validate the associations in the primary analysis, we conducted sensitivity analyses, including MR-heterogeneity [29], MR-pleiotropy, MR-Pleiotropy RESidual Sum and Outlier (PRESSO), and colocalization analysis [30]. MR-heterogeneity (*p* < 0.05) or Colocalization analysis (PP.H4.abf <80%) suggested the presence of heterogeneity. MR-pleiotropy (*p* < 0.05) suggested the presence of pleiotropy. We tested the effects of proteins on MI, and then the effects of potential mediations (body mass index (BMI), fasting blood glucose (FBG), glycosylated hemoglobin, type A1C (HbA1c), low-density lipoprotein (LDL), high-density lipoprotein (HDL), and triglyceride (TG)) using two-step MR [31].

### 2.5 Phe-MR Analysis

We evaluated potential on-target side effects associated with interventions targeting identified proteins to reduce MI burden, using summary statistics from the FinnGen biobank’s GWAS analysis of 2803 disease traits (https://gwas.mrcieu.ac.uk/). In this study, representative traits were exclusively chosen to minimize inherent redundancy and, consequently, enhance the quality of the results. Furthermore, exclusion criteria were applied for sex-specific disease traits, disease traits with similar profiles, and disease traits with fewer than 500 cases, respectively. This was done to account for data availability and statistical significance issues. Finally, the Phe-MR analysis included 310 non-MI disease traits to explore potential on-target side effects associated with proteins related to MI (Fig. 1; **Supplementary Table 2**).

In the second stage, a statistically significant association was considered when the observed 2-sided *p*-value was below 1.51 × ${\mathrm{10}}^{-4}$ (Bonferroni-corrected: *p* = 0.05/331). For stage four, the established threshold for statistical significance in the Phe-MR analysis was *p* = 0.05/1550 (resulting from the multiplication of 5 identified MI proteins in stage two by 310 diseases) = 3.23 × ${\mathrm{10}}^{-5}$ (Bonferroni-corrected). R software (version 4.2.2, R Foundation for Statistical Computing, Vienna, Austria) was employed for all statistical analyses, utilizing packages such as gtx, MendelianRandomization, TwoSampleMR, ggplot2, dplyr, qqman, ggrepel, CMplot, forestploter, coloc, ieugwasr, gwasvcf, gwasglue, and VariantAnnotation.

## 3. Results

### 3.1 Strength of the Genetic Instruments for Blood Proteins

In this MR analysis, a total of 331 blood proteins were examined (**Supplementary Table 3**). The genetic instruments accounted for variance in the proteins ranging from 0.87% to 10.32%. The genetic instruments for the proteins exhibited F statistics ranging from 29.67 to 9926.96, indicating the absence of weak instrument bias (**Supplementary Table 3**).

### 3.2 Identification of Causal Proteins for MI from the Blood Proteome

**Fig. 2.**
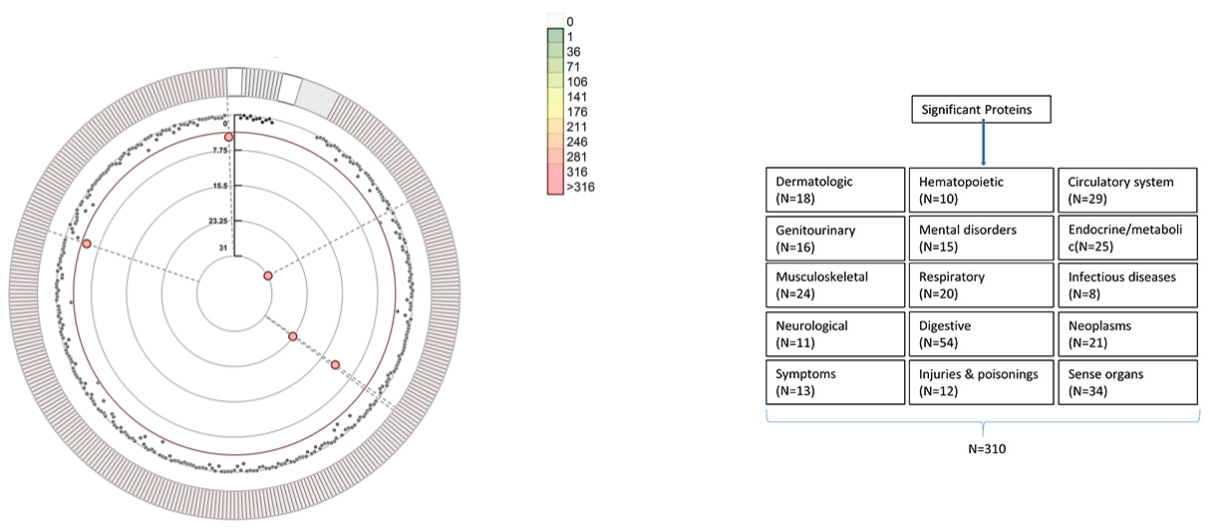
**Circular Manhattan plot illustrating the associations between blood proteins and the risk of myocardial infarction (MI).** The Bonferroni-corrected significance threshold (*p* < 0.000151) is depicted by the dashed line, with labels indicating significant proteins. The 331 proteins are grouped and color-coded based on sample size. Results for the associations between proteins and MI can be found in **Supplementary Table 5**.

The primary MR analysis investigated the relationships between the risk of MI and 331 blood proteins (**Supplementary Table 4**). In the principal analysis, genetically determined cardiotrophin-1 (CT-1), Selenoprotein S (SELENOS), killer cell immunoglobulin-like receptor 2DS2 (KIR2DS2), vacuolar protein sorting-associated protein 29 (VPS29), and histo-blood group ABO system transferase (NAGAT) demonstrated significant associations with an elevated risk of MI (Fig. 2 and **Supplementary Table 5**). Following this, sensitivity analyses including MR-heterogeneity, MR-pleiotropy, colocalization analysis, and MR-PRESSO were performed, as shown in **Supplementary Table 6** and **Supplementary Table 7**.

In total, 5 proteins with causal implications for the risk of MI were identified (Table 2 and **Supplementary Table 7**). For these proteins, each standard deviation (SD) increase in genetically determined KIR2DS2 was associated with a lower risk of MI (OR: 0.93; 95% CI: 0.90–0.96; *p* = 1.08 × ${\mathrm{10}}^{-5}$), along with VPS29 (OR: 0.92; 95% CI: 0.90–0.94; *p* = 8.05 × ${\mathrm{10}}^{-13}$). In contrast, each SD increase in genetically determined CT-1 (OR: 1.16; 95% CI: 1.13–1.18; *p* = 1.29 × ${\mathrm{10}}^{-31}$), SELENOS (OR: 1.16; 95% CI: 1.13–1.20; *p* = 4.73 × ${\mathrm{10}}^{-24}$), and NAGAT (OR: 1.05; 95% CI: 1.03–1.07; *p* = 1.41 × ${\mathrm{10}}^{-5}$) was associated with a higher risk of MI.

**Table 2.** **Summary of significant human blood proteins representing causal mediators for MI**.

Proteins	SNPs	Methods	beta	se	OR	95% CI	*p* value
CT-1	3	MR Egger	0.110	0.193	1.116	0.765–1.629	0.6709316
		Weighted median	0.145	0.014	1.156	1.125–1.188	2.2435 × ${\mathrm{10}}^{–\,25}$
		IVW	0.145	0.012	1.156	1.128–1.184	1.2876 × ${\mathrm{10}}^{–\,31}$
		Simple mode	0.177	0.027	1.194	1.133–1.258	0.02213021
		Weighted mode	0.135	0.014	1.145	1.113–1.178	0.01129431
SELENOS	3	MR Egger	0.096	0.041	1.101	1.016–1.194	0.256226
		Weighted median	0.149	0.016	1.160	1.125–1.197	1.1878 × ${\mathrm{10}}^{–\,20}$
		IVW	0.152	0.015	1.164	1.131–1.199	4.7338 × ${\mathrm{10}}^{–\,24}$
		Simple mode	0.161	0.032	1.175	1.103–1.251	0.03786816
		Weighted mode	0.147	0.016	1.158	1.122–1.196	0.01214628
KIR2DS2	6	MR Egger	–0.124	0.037	0.883	0.822–0.949	0.02764757
		Weighted median	–0.093	0.020	0.911	0.877–0.947	2.3888 × ${\mathrm{10}}^{–\,06}$
		IVW	–0.075	0.017	0.927	0.897–0.959	1.0796 × ${\mathrm{10}}^{–\,05}$
		Simple mode	–0.090	0.036	0.914	0.852–0.980	0.05255789
		Weighted mode	–0.093	0.021	0.911	0.873–0.950	0.00730637
VPS29	3	MR Egger	–0.096	0.011	0.909	0.889–0.928	0.07263406
		Weighted median	–0.086	0.009	0.917	0.901–0.934	6.7061 × ${\mathrm{10}}^{–\,22}$
		IVW	–0.084	0.012	0.919	0.899–0.941	8.0535 × ${\mathrm{10}}^{–\,13}$
		Simple mode	–0.091	0.023	0.913	0.873–0.954	0.05643502
		Weighted mode	–0.087	0.009	0.917	0.900–0.934	0.01171093
NAGAT	3	MR Egger	0.065	0.022	1.067	1.023–1.113	0.2048195
		Weighted median	0.048	0.006	1.050	1.038–1.062	2.4082 × ${\mathrm{10}}^{–\,16}$
		IVW	0.048	0.011	1.049	1.026–1.072	1.4119 × ${\mathrm{10}}^{–\,05}$
		Simple mode	0.018	0.029	1.018	0.962–1.077	0.5990393
		Weighted mode	0.050	0.006	1.052	1.039–1.065	0.01494713

Association estimates with the risk of MI per 1-SD increase in CT-1, SELENOS, VPS29, KIR2DS2, and NAGAT are represented by ORs along with their 95% CIs. The significant threshold was established at *p* < 0.000151. CT-1, cardiotrophin-1; SELENOS, Selenoprotein S; KIR2DS2, killer cell immunoglobulin-like receptor 2DS2; VPS29, vacuolar protein sorting-associated protein 29; NAGAT, histo-blood group ABO system transferase; MI, myocardial infarction; SNPs, single nucleotide polymorphisms; OR, odds ratio; CI, confidence interval; MR, mendelian randomization; IVW, inverse-variance weighted.

### 3.3 Identification of Potential MI Risk Factors

To determine potential mechanisms linking 5 plasma proteins and MI, a two-step mediation MR analysis was conducted for conventional MI risk factors. Initially, two-sample MR analyses were performed to delineate the causal relationships between the MI risk factors and MI itself. The correlation between the screened 5 proteins which we identified from the GWAS and the conventional risk factors of MI were assessed subsequently. For each of the 6 considered MI risk factors (i.e., BMI, FBG, HbA1c, LDL, HDL, and TG). Notably, BMI, HbA1c, LDL, and TG were linked to an increased MI risk, whereas HDL was linked to a decreased MI risk (*p* ≤ 0.05/6 = 0.0083, Bonferroni adjusted for 6 risk factors). No significant association was found between FBG and MI (*p* > 0.05) (**Supplementary Table 8**).

We performed MR of 5 significant Proteins with the 5 MI risk factors (BMI, HbA1c, LDL, HDL, TG). Out of the 5 proteins associated with MI, 4 were identified to be linked with one or more of the risk factors for MI (**Supplementary Table 9**, **Supplementary Fig. 1**). BMI and HDL were associated with lower CT-1 levels while LDL was associated with higher CT-1 levels (*p* ≤ 0.05/(5 × 5) = 0.002). LDL was associated with higher SELENOS levels while HDL was associated with lower SELENOS levels (*p* ≤ 0.002). HDL and TG was associated with higher VPS29 levels while LDL was associated with lower VPS29 levels (*p* ≤ 0.002). HDL and LDL was associated with higher NAGAT levels while TG was associated with lower NAGAT levels (*p* ≤ 0.002).

To determine the indirect impact of proteins on MI outcomes through risk factors, a mediation analysis was conducted, utilizing the effect estimates derived from the two-step MR and the total effect from the primary MR (**Supplementary Table 10**). The mediator factors were screened by causality relationship analysis among the potential mediators with outcome of MI after the exposure of the five identified proteins. (**Supplementary Tables 8,9**). The ideal mediator variables are defined by the *p*-value < 0.05 with the horizontal pleiotropy >0.05, which calculated by IVW and weighted-median methods. it can be observed that BMI and TG meet these criteria. The CT-1-MI effect remained non-significantly altered, ranging from 1.156 (95% CI 1.13, 1.18) to 1.158 (95% CI 1.11, 1.20), after adjusting for the estimated effects of BMI. The SELENOS–MI effect reduced from 1.16 (95% CI 1.13, 1.29) to 1.14 (95% CI 1.09, 1.20) after adjusting for the estimated effects of BMI. The VPS29-MI effect increased from 0.92 (95% CI 0.90, 0.94) to 0.93 (95% CI 0.90, 0.96) after adjusting for the estimated effects of BMI and triacylglycerols. The NAGAT-MI effect reduced from 1.05 (95% CI 1.03, 1.07) to 1.04 (95% CI 1.02, 1.07) with adjustment for the estimated effects of triacylglycerols. However, after adjusting for triacylglycerols, the causal link between KIR2DS2 and MI dissipated (*p* > 0.05).

### 3.4 Phe-MR Analysis

**Fig. 3.**
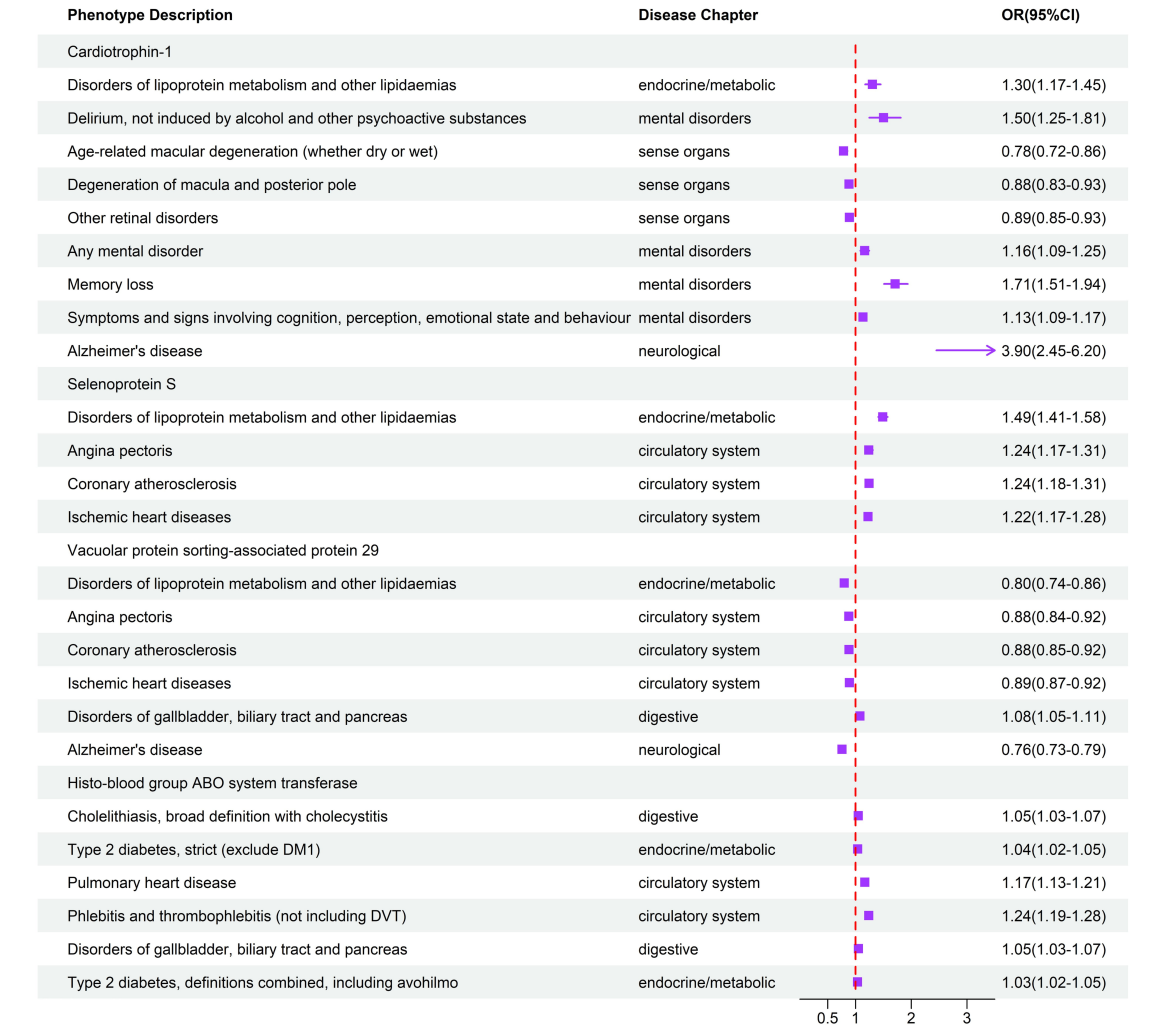
**Potential on-target side effects associated with CT-1, SELENOS, VPS29, and NAGAT intervention revealed by Phe-MR analysis.** Effect estimates on the risk of multiple non-MI per 10% reduction in risk for MI, achieved by targeting CT-1, SELENOS, VPS29, and NAGAT, are presented as ORs along with their 95% CIs. CT-1, cardiotrophin-1; SELENOS, Selenoprotein S; VPS29, vacuolar protein sorting-associated protein 29; NAGAT, histo-blood group ABO system transferase; MI, myocardial infarction; OR, odds ratio; CI, confidence interval; MR, mendelian randomization; Phe-MR, phenome-wide MR; DVT, deep venous thrombosis; DM1, type 1 diabetes mellitus.

A comprehensive Phe-MR analysis was employed to systematically evaluate the effects of the identified MI proteins on the risks associated with 310 non-MI diseases (**Supplementary Table 2**), aiming to elucidate their potential side-effect profiles. A total of 25 associations reached a threshold of *p* = 3.23 × ${\mathrm{10}}^{-5}$ (calculated as 0.05/1550 [5 proteins × 310 diseases]) (**Supplementary Tables 11,12,13,14,15**, Fig. 3, **Supplementary Figs. 2,3,4,5,6**). The results from sensitivity analyses, incorporating heterogeneity and pleiotropy, provided additional validation for the associations identified in the Phe-MR analysis (**Supplementary Table 16**).

Targeting the CT-1, SELENOS, VPS29, and NAGAT revealed a total of 25 significant associations with various non-MI diseases (Fig. 3 and **Supplementary Table 17**). In brief, CT-1 had detrimental effects on 4 mental disorders diseases (memory loss; symptoms and signs involving cognition, perception, emotional state and behavior; any mental disorder; Delirium), 1 neurological system disease (Alzheimer’s disease), and 1 endocrine/metabolic disease (disorders of lipoprotein metabolism and other lipidemias), while it had beneficial effects on 3 sense organs disorders (Age-related macular degeneration; Degeneration of macula and posterior pole; Other retinal disorders). SELENOS exhibited harmful effects on 3 circulatory system diseases (Angina pectoris; Coronary atherosclerosis; ischemic heart diseases) and 1 endocrine/metabolic disease (Disorders of lipoprotein metabolism and other lipidemias). VPS29 had beneficial effects on 3 circulatory system diseases (Angina pectoris; Coronary atherosclerosis; ischemic heart diseases), 1 neurological system disease (Alzheimer’s disease), and 1 endocrine/metabolic disease (Disorders of lipoprotein metabolism and other lipidemias), while it had detrimental effects on 1 digestive system disease (Disorders of gallbladder, biliary tract and pancreas). In addition, NAGAT had detrimental effects on 2 circulatory system diseases (Phlebitis and thrombophlebitis (not including deep venous thrombosis (DVT)); Pulmonary heart disease), 2 endocrine/metabolic disease which were associated with type 2 diabetes, and 2 digestive system diseases (Cholelithiasis; Disorders of gallbladder, biliary tract and pancreas).

The associations with the most significant impact were observed in Memory loss (OR per 10% reduction in MI risk: 1.71; 95% CI: 1.51–1.94; *p* = 2.06 × ${\mathrm{10}}^{-17}$) for CT-1, Disorders of lipoprotein metabolism and other lipidaemias (OR per 10% reduction in MI risk: 1.49; 95% CI: 1.41–1.58; *p* = 2.91 × ${\mathrm{10}}^{-43}$) for SELENOS, Alzheimer’s disease (OR per 10% reduction in MI risk: 0.76; 95% CI: 0.73–0.79; *p* = 2.52 × ${\mathrm{10}}^{-54}$) for VPS29, and Phlebitis and thrombophlebitis (not including DVT) (OR per 10% reduction in MI risk: 1.24; 95% CI: 1.19–1.28; *p* = 2.22 × ${\mathrm{10}}^{-28}$) for NAGAT (**Supplementary Table 16**).

## 4. Discussion

By integrating genomics with proteins, the current MR study has offered novel insights into the exploration of promising and safe drug targets for MI. Out of 331 blood proteins examined, we identified 5 proteins with potential causal associations with MI: CT-1, SELENOS, KIR2DS2, VPS29, and NAGAT. Among the 5 identified proteins, KIR2DS2 and VPS29 had protective effects, while CT-1, SELENOS, and NAGAT had detrimental effects on MI. The Phe-MR analysis was carried out to anticipate on-target side effects linked to potential MI treatment through interventions targeting the identified proteins. It was observed that CT-1 exerted adverse effects on 4 mental disorders, 1 neurological system disease, and 1 endocrine/metabolic disease, while it had beneficial effects on 3 sense organs disorders. NAGAT had detrimental effects on 2 circulatory system diseases, 2 endocrine/metabolic diseases which were associated with type 2 diabetes, and 2 digestive system disease. SELENOS had detrimental effects on 3 circulatory system diseases and 1 endocrine/metabolic disease, while VPS29 had beneficial effects on these diseases and 1 neurological system disease. VPS29 had detrimental effects on 1 digestive system disease.

As a member of the gp130 family of cytokines, CT-1 is known for its diverse physiological roles [32]. CT-1 was a key factor in cardiomyocyte maturation and promoted cell survival of serum-deprived neonatal rat cardiomyocytes through mitogen-activated protein kinase (MAPK) and extracellular regulated protein kinases (ERK)1/2 mediated anti-apoptotic pathways [33]. In rats experiencing MI, ischemic heart disease, valvular heart disease, and post-MI conditions, there was an increase in both the mRNA and protein levels of CT-1 [34]. Freed *et al*. [35] demonstrated that CT-1 fosters the formation of infarct scars by upholding the cellular structure of the scar, consequently enhancing ventricular function. Notably, CT-1 exhibited the ability to restrict myocardial injury even when administered during reoxygenation. Beyond its impacts on the heart, CT-1 exerts significant protective effects on various organs, including the liver, kidneys, and nervous system. Numerous studies have indicated that CT-1 may play a pivotal role in regulating body weight and metabolism [32, 36, 37, 38]. In our study, elevated levels of CT-1 were correlated with a lower BMI and HDL, along with an increased risk of LDL. Analyzing data from a MI GWAS involving 638,717 European participants, our findings revealed a genetically determined higher blood level of CT-1 associated with an increased risk of MI. This suggests that increased levels of CT-1 are linked to a heightened risk of MI. Furthermore, the Phe-MR analysis indicated that CT-1 exhibited adverse effects on four mental disorders, 1 neurological system disease, and 1 endocrine/metabolic disease, while it had beneficial effects on 3 sense organs disorders. Hence, considering the identification of certain adverse side effects through Phe-MR analysis, the application of a therapeutic strategy involving CT-1 for MI prevention and treatment should be approached after a careful evaluation of the pros and cons associated with CT-1.

Excessive inflammation plays a pivotal role in triggering and contributing to cardiovascular disease, making it a significant therapeutic target [39, 40]. The identification of novel biomarkers has enriched our understanding of inflammation, complementing established indicators such as C-reactive protein, interleukins (ILs), and tumor necrosis factor alpha. In a study by Sardu *et al*. [39], sirtuins, microRNAs, suppression of tumorigenicity 2 (ST2) protein, apolipoprotein E protein, and adiponectin emerged as promising biomarkers for the diagnosis and prognosis of cardiovascular disease (CVD). Moreover, these newly identified inflammatory biomarkers offer valuable insights into evaluating the efficacy of treatments in patients with CVD. A study revealed that hyperglycemic ST-elevated myocardial infarction (STEMI) patients, in contrast to their normoglycemic counterparts undergoing thrombus aspiration treatment, exhibited elevated levels of pro-inflammatory cytokines, specifically tumor necrosis factor-alpha, within coronary artery thrombi [40]. Regarding glycemic control, sodium-dependent glucose transporters 2 (SGLT2) inhibitors exhibit the potential to induce a more stable phenotype in coronary atherosclerotic plaques, as evidenced by increased minimum fibrous cap thickness and reduced inflammation and lipid deposition. This beneficial effect is attributed to the improvement of glucose homeostasis and the attenuation of systemic inflammatory burden, as indicated by decreased levels of NLR family pyrin domain containing 3 (NLRP3), serum caspase-1, and IL-1β [41].

As participants in the regulation of inflammation and oxidative stress, SELENOS operates as a member of the selenoprotein family [42, 43, 44, 45, 46]. Recent studies have revealed novel histological distributions of SELENOS in the spleen, blood vessels, and serum [47, 48]. Alanne *et al*. [49] found a higher risk of cardiovascular disease among SELENOS SNP rs8025174 carriers in women (hazard ratio 2.95). In their analysis of the association between 10 types of SELENOS gene polymorphisms and the risk of atherosclerosis in type 2 diabetes mellitus (T2DM) patients, Cox *et al*. [50] identified associations between SELENOS SNPs and both subclinical and clinical atherosclerosis. In our study, an association was observed in which elevated levels of SELENOS were linked to lower HDL and an increased risk of LDL. Vascular endothelial cells, and vascular smooth muscle cells (VSMCs) are crucial for cardiovascular homeostasis [51]. A study demonstrated that heightened SELENOS expression enhanced human umbilical vein endothelial cells (HUVEC) viability and superoxide dismutase activity while reducing $H_{2}$$O_{2}$-induced malondialdehyde production [52]. Another study also found that inhibiting SELENOS expression in VSMCs exacerbated cell damage induced by $H_{2}$$O_{2}$ or tunicamycin and increased VSMC apoptosis. These results indicated that SELENOS could increase the resistance of HUVECs and VSMCs to oxidative stress [48]. In this MR study, a positive association was identified between genetically determined levels of SELENOS and the risk of MI. Considering the identification of certain adverse side effects through Phe-MR analysis, the application of a therapeutic strategy involving SELENOS for MI prevention and treatment should be approached after a careful evaluation of the pros and cons associated with SELENOS.

KIR2DS2 was one of killer immunoglobulin-like receptors (KIRs) [53]. Studies reported that there was a higher prevalence of certain activators KIR gene (2DS2 and 2DS4) in subjects with acute ischemic stroke [54], acute coronary syndrome [55, 56] and unstable atherosclerotic plaques [57]. In patients with rheumatoid vasculitis [58] and acute coronary syndrome [59], CD4+CD28-T cells expressing KIR2DS2 in the absence of opposing inhibitory receptors may promote the activation of autoreactive T cells linked to the mechanisms responsible for the instability of atherosclerotic plaques and ischemic neuronal damage. Although the relationship between KIR2DS2 and MI has not been reported, our results suggest that genetically determined higher KIR2DS2 levels are linked to a reduced risk of MI. Consequently, KIR2DS2 may emerge as a promising drug target for preventing and treating MI, devoid of predicted harmful side effects.

Operating within the endolysosomal pathway, the protein complex Retromer, which encompasses VPS35, VPS26, and VPS29, is responsible for the recycling of proteins. Playing a central role as a scaffold, VPS29 coordinates the assembly of retrieval complexes with regulatory components [60]. Research utilizing genetic, cellular, and animal models has linked retrotransposons and their interacting proteins to familial neurodegenerative diseases. Although no relationship between VPS29 and cardiovascular disease has been reported, VPS29 has been shown to be associated with aging. In the investigation conducted by Chu and Praticò [61], it was discovered that the primary components of the retromer recognition core experienced a significant reduction with age in the brain cortices of Tg2576 mice. Aging, a crucial risk factor in the development of MI, is considered to involve the loss of protein homeostasis, a shared characteristic in the pathogenesis of MI. In our investigation, an association was observed between VPS29 and lipid levels, implying that the pathways by which VPS29 influences MI risk may be connected to lipid levels. Further research is required to determine the exact mechanism. Our results suggest that genetically determined elevated VPS29 levels are linked to a reduced risk of MI. Furthermore, the Phe-MR analysis confirmed the previously observed beneficial role of VPS29 in the circulatory system, suggesting it as a novel and promising drug target for preventing and treating MI, with additional protective effects against 5 diseases.

NAGAT lies at the core of the ABO blood group system. This system encompasses 3 carbohydrate antigens: A, B, and H. NAGAT was closely associated with cancer [62]. A MR Analysis about Ischemic Stroke showed that NAGAT was a causal mediator for cardioembolic stroke [24]. In our investigation, an association was established between genetically determined higher NAGAT levels and an increased MI risk, suggesting that elevated levels of NAGAT are linked to an elevated risk of MI. Additionally, our findings demonstrated that higher levels of NAGAT were correlated with elevated LDL. The exact mechanism is unclear. Phe-MR analysis also showed that NAGAT has a detrimental effects on 2 circulatory system diseases, 2 endocrine/metabolic diseases which were associated with type 2 diabetes, and 2 digestive system diseases. Consequently, the application of a therapeutic strategy involving NAGAT for the prevention and treatment of MI should be considered after a thorough evaluation of the pros and cons associated with NAGAT.

The implications of our findings are significant for both public health and clinical practice. MI is as a prominent cause of global mortality. Coronary atherosclerosis, characterized by stable and unstable periods well before the manifestation of overt symptoms, provides a substantial time window for interventions to delay the disease’s progression [23]. Hence, it holds paramount importance to identify key biomarkers that can pinpoint individuals at a heightened risk for circulatory diseases to facilitate the early prevention of MI. The results indicate that certain blood proteins (CT-1, SELENOS, KIR2DS2, VPS29, and NAGAT) have the potential to serve as predictive biomarkers for MI. However, recently, two studies on MR of MI have yielded different findings. Wu *et al*. [63] identified two proteins, lipoprotein(a) (LPA) and apolipoprotein A5 (APOA5), as potential drug targets for MI, with their causal effects on MI risk. Ye *et al*. [64] identified seven promising drug targets for intervention in MI (switch-associated protein 70 (SWP70), transgelin-2 (TAGLN2), low-density lipoprotein receptor-related protein 4 (LRP4), C1s subcomponent (C1s), apolipoprotein C-III (Apo C-III), proprotein convertase subtilisin/kexin type 9 (PCSK9), and angiopoietin-related protein 4 (ANGL4)). The difference in results may be due to the different databases used. Therefore, our study proposes new drug targets that can complement these two studies. As biomarker testing becomes more comprehensive, the discovery of numerous additional drug targets may be discovered. Phe-MR results indicate that biomarkers influencing MI may mediate the risk of numerous non-MI diseases, involving both circulatory and non-circulatory disorders. Notably, certain biomarkers (KIR2DS2, VPS29) predicted the occurrence of potential side-effects which would be beneficial for drug development.

Several factors limit the interpretation and generalizability of the study findings. First, while the MR study incorporated 331 diverse proteins from 3 extensive GWASs through stringent selection criteria, it’s important to note that these proteins represent only a fraction of the total blood proteins. Second, the participants enrolled in this study were exclusively of European ancestry. Although this choice minimizes the potential for spurious associations stemming from population selection bias, it does impose a constraint on extrapolating our findings to non-European populations. Future biomarker GWAS in non-European cohorts are imperative to facilitate trans-ethnic MR analyses, expected to yield more broadly applicable findings. Third, our study data did not include the latest protein GWASs [65, 66, 67] and MI GWAS [68]. Therefore, a further and more comprehensive exploration of potential drug targets for MI is still warranted.

## 5. Conclusions

In our comprehensive MR study, we employed a systematic approach to unravel the intricate relationships between genetically predicted elevations in specific 5 plasma proteins and the risk of incident MI. Our findings shed light on distinct patterns of association, offering valuable insights for potential interventions and drug targeting. Genetically elevated levels of KIR2DS2 and VPS29 are linked to a decreased risk of MI. Conversely, CT-1, SELENOS, and NAGAT are associated with an increased risk of MI. Notably, KIR2DS2 stands out as a promising target for MI prevention and treatment, with no side effects. Furthermore, VPS29 could also be a viable option. Despite its detrimental effects on 1 digestive system disease, its overall beneficial effects on 5 other diseases underscore its potential as a multifaceted therapeutic target. Further research into the specific mechanisms and pathways involved will be crucial for a more nuanced understanding of these associations and to guide future clinical applications.

## Data Availability

The data further inquiries can be directed to the corresponding author.

## Supplementary Material

Supplementary material associated with this article can be found, in the online version, at https://doi.org/10.31083/j.rcm2506199.
