# Supplementary material for: Association between Human Blood Proteome and the Risk of Myocardial Infarction
Source: Rev Cardiovasc Med. 2024 May 30;25(6):199. doi: 10.31083/j.rcm2506199 (PMC11270110; doi:10.31083/j.rcm2506199)
Supplement: Supplementary file 1 [file 2153-8174-25-6-199-s1.zip › Supplementary figures.docx]

**Summary of supplementary tables and figures**

Table s1 Description of Datasets Used for GWAS Meta-analysis for MI

Table s2. 310 diseases incorporated in the Phe-MR analysis

Table s3. Overview of the Blood Proteins included in the MR study

Variance：Phenotypic variance in each blood protein explained by used genetic variants in the present MR study. F value：The strength of genetic instruments for each blood protein in the present MR study

Table s4 MR analysis for the associations between blood proteins and MI.

Odds ratios (ORs) with their 95% confidence intervals (CIs) represent the association estimates with the risks of MI of per 1– standard deviation increase in blood protein levels. Significant threshold was set at P<0.000151 (Bonferroni-corrected significance threshold calculated as 0.05 divided by 331 [for 331 blood proteins]).

Table s5 IVW MR analysis for the associations between blood proteins and MI

Odds ratios (ORs) with their 95% confidence intervals (CIs) represent the association estimates with the risks of MI of per 1– standard deviation increase in blood protein levels. Significant threshold was set at P<0.000151 (Bonferroni-corrected significance threshold calculated as 0.05 divided by 331 [for 331 blood proteins]).

Table s6 Sensitivity analyses and colocalization analysis for blood proteins

Odds ratios (ORs) with their 95% confidence intervals (CIs) represent the association estimates with the risk of MI of per 1– standard deviation increase in blood protein levels.

Significant threshold was set at P<0.000151 (Bonferroni-corrected significance threshold calculated as 0.05 divided by 331 [for 331 blood proteins]).

Table s7 Sensitivity analyses and colocalization analysis for significant blood proteins

Odds ratios (ORs) with their 95% confidence intervals (CIs) represent the association estimates with the risk of MI of per 1– standard deviation increase in CT-1, SELENOS, VPS29, KIR2DS2, and NAGAT.

Significant threshold was set at P<0.000151 (Bonferroni-corrected significance threshold calculated as 0.05 divided by 331 [for 331 blood proteins]).

Table s8 MR estimates of cardiovascular risk factors on MI

Abbreviations: BMI, body mass index; FBG, fasting blood glucose; HbA1c, glycosylated hemoglobin; LDL, low density lipoprotein; HDL,high density lipoprotein; TG, triglyceride.

Table s9 MR estimates of significant human blood proteins on cardiovascular risk factors

Abbreviations: BMI, body mass index; FBG, fasting blood glucose; HbA1c, glycosylated hemoglobin; LDL, low density lipoprotein; HDL,high density lipoprotein; TG, triglyceride.

Table s10 Multivariate separate-sample MR analysis of the effect of Significant Human Blood Proteins on MI

Abbreviations: BMI, body mass index; TG, triglyceride.

Table s11. Phe-MR analyses for the associations between CT-1 and 310 diseases using the IVW method

Odds ratios (ORs) with their 95% confidence intervals (CIs) represent the effect estimates on the risk of multiple non-MI of per 10% reduction in risk for MI by targeting CT-1.

Significant threshold was set at P<3.23×10-5 (Bonferroni-corrected significance threshold calculated as 0.05 divided by 1550 [5 proteins ×310 diseases]).

Table s12. Phe-MR analyses for the associations between SELENOS and 310 diseases using the IVW method

Odds ratios (ORs) with their 95% confidence intervals (CIs) represent the effect estimates on the risk of multiple non-MI of per 10% reduction in risk for MI by targeting SELENOS.

Significant threshold was set at P<3.23×10-5 (Bonferroni-corrected significance threshold calculated as 0.05 divided by 1550 [5 proteins ×310 diseases]).

Table s13. Phe-MR analyses for the associations between VPS29 and 310 diseases using the IVW method

Odds ratios (ORs) with their 95% confidence intervals (CIs) represent the effect estimates on the risk of multiple non-MI of per 10% reduction in risk for MI by targeting VPS29.

Significant threshold was set at P<3.23×10-5 (Bonferroni-corrected significance threshold calculated as 0.05 divided by 1550 [5 proteins ×310 diseases]).

Table s14. Phe-MR analyses for the associations between KIR2DS2 and 310 diseases using the IVW method

Odds ratios (ORs) with their 95% confidence intervals (CIs) represent the effect estimates on the risk of multiple non-MI of per 10% reduction in risk for MI by targeting KIR2DS2.

Significant threshold was set at P<3.23×10-5 (Bonferroni-corrected significance threshold calculated as 0.05 divided by 1550 [5 proteins ×310 diseases]).

Table s15. Phe-MR analyses for the associations between NAGAT and 310 diseases using the IVW method

Odds ratios (ORs) with their 95% confidence intervals (CIs) represent the effect estimates on the risk of multiple non-MI of per 10% reduction in risk for MI by targeting NAGAT.

Significant threshold was set at P<3.23×10-5 (Bonferroni-corrected significance threshold calculated as 0.05 divided by 1550 [5 proteins ×310 diseases]).

Table s16.Phe-MR analyses for causal associations of CT-1, SELENOS, VPS29, and NAGAT with the risk of multiple non-MI diseases

Odds ratios (ORs) with their 95% confidence intervals (CIs) represent the effect estimates on the risk of multiple non-MI of per 10% reduction in risk for MI by targeting CT-1, SELENOS, VPS29, and NAGAT, respectively.

Significant threshold was set at P<3.23×10-5 (Bonferroni-corrected significance threshold calculated as 0.05 divided by 1550 [5 proteins ×310 diseases]).

Table s17. Summary of significant Phe-MR findings representing side effects associated with targeting identified proteins

Significant threshold was set at P<3.23×10-5 (Bonferroni-corrected significance threshold calculated as 0.05 divided by 1550 [5 proteins ×310 diseases]).

Figure s1 Correlation coefficient (beta) of five potential causal proteins on causal risk factors for MI

Colours in each lattice of the heatmap represent the correlation coefficient (beta). Red color represents a positive correlation, blue color represents a negative correlation. The darker the color the larger the effect size.

Figure s2 The Manhattan plot displaying on-target side effects of CT-1 in the IVW Phe-MR analysis

The dashed line represents the Bonferroni-corrected significance threshold (P<3.23×10-5 ), and the labels are provided for significant diseases. The 310 diseases are grouped and color-coded by Disease Chapter listed in Table S11. The detailed results for the associations between diseases and CT-1 by IVW Mendelian randomization analysis are presented in Table S11.

Figure s3 The Manhattan plot displaying on-target side effects of SELENOS in the IVW Phe-MR analysis

The dashed line represents the Bonferroni-corrected significance threshold (P<3.23×10-5 ), and the labels are provided for significant diseases. The 310 diseases are grouped and color-coded by Disease Chapter listed in Table S12. The detailed results for the associations between diseases and SELENOS by IVW Mendelian randomization analysis are presented in Table S12.

Figure s4 The Manhattan plot displaying on-target side effects of VPS29 in the IVW Phe-MR analysis

The dashed line represents the Bonferroni-corrected significance threshold (P<3.23×10-5 ), and the labels are provided for significant diseases. The 310 diseases are grouped and color-coded by Disease Chapter listed in Table S13. The detailed results for the associations between diseases and VPS29 by IVW Mendelian randomization analysis are presented in Table S13.

Figure s5 The Manhattan plot displaying on-target side effects of KIR2DS2 in the IVW Phe-MR analysis

The detailed results for the associations between diseases and KIR2DS2 by IVW Mendelian randomization analysis are presented in Table S14.

Figure s6 The Manhattan plot displaying on-target side effects of NAGAT in the IVW Phe-MR analysis

The dashed line represents the Bonferroni-corrected significance threshold (P<3.23×10-5), and the labels are provided for significant diseases. The 310 diseases are grouped and color-coded by Disease Chapter listed in Table S15. The detailed results for the associations between diseases and NAGAT by IVW Mendelian randomization analysis are presented in Table S15.


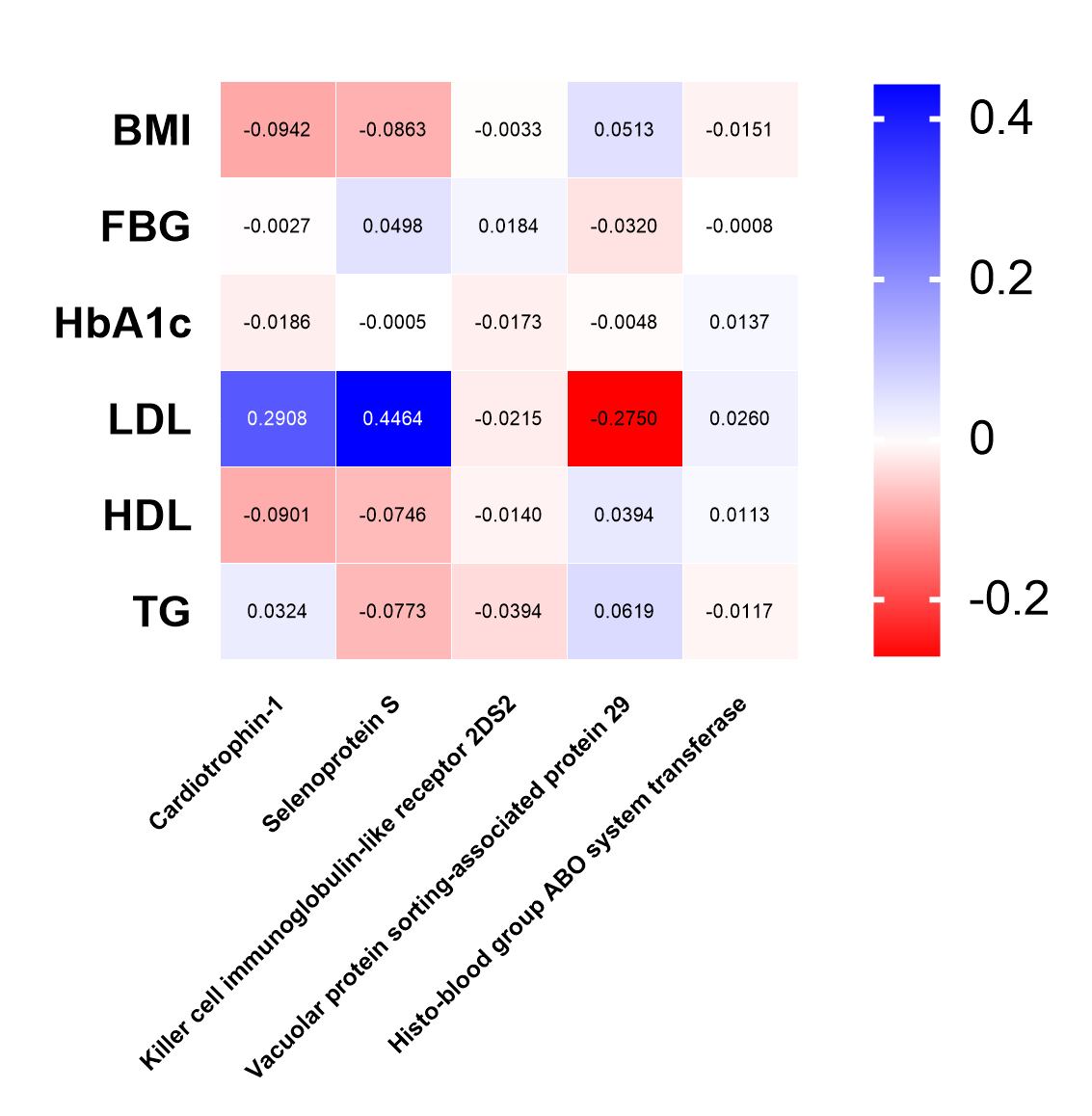


CT-1

SELENOS

KIR2DS2

NAGAT

VPS29

BMI

FBG

HbA1C

LDL

HDL

TG

0

-0.2

0.2

0.4

Figure S1 Correlation coefficient (beta) of five potential causal proteins on causal risk factors for MI


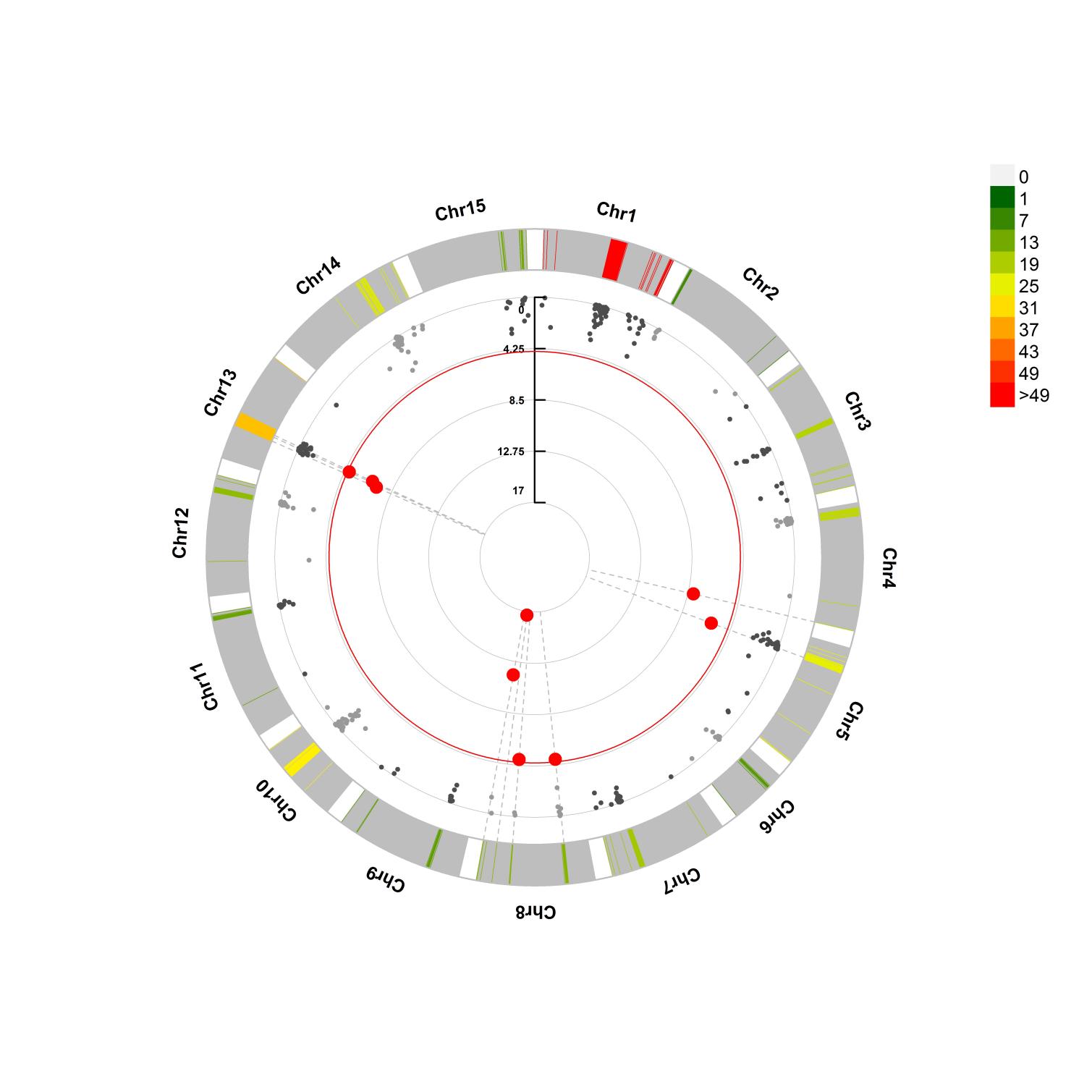


Figure S2 The Manhattan plot displaying on-target side effects of CT-1 in the IVW Phe-MR analysis


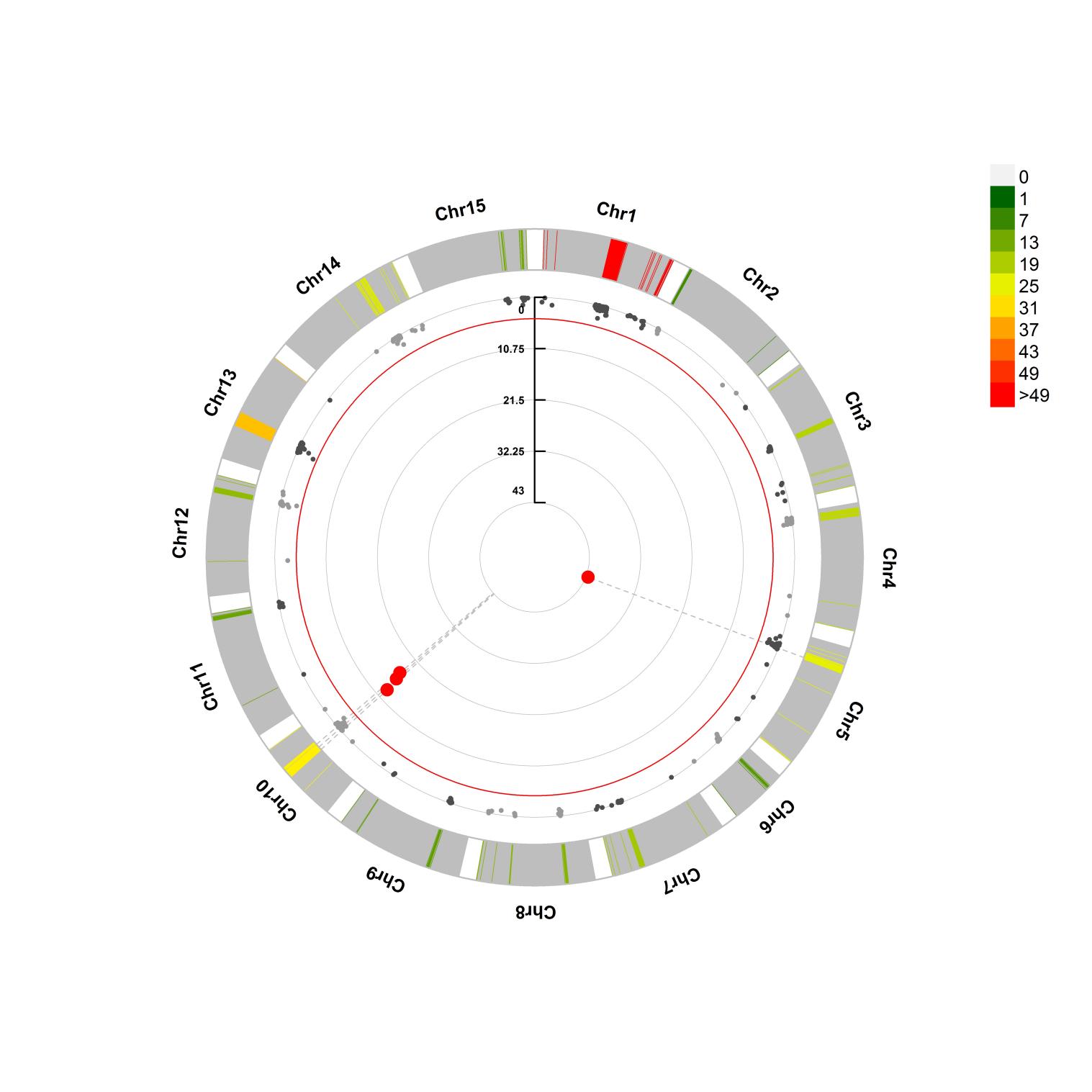


Figure S3 The Manhattan plot displaying on-target side effects of SELENOS in the IVW Phe-MR analysis


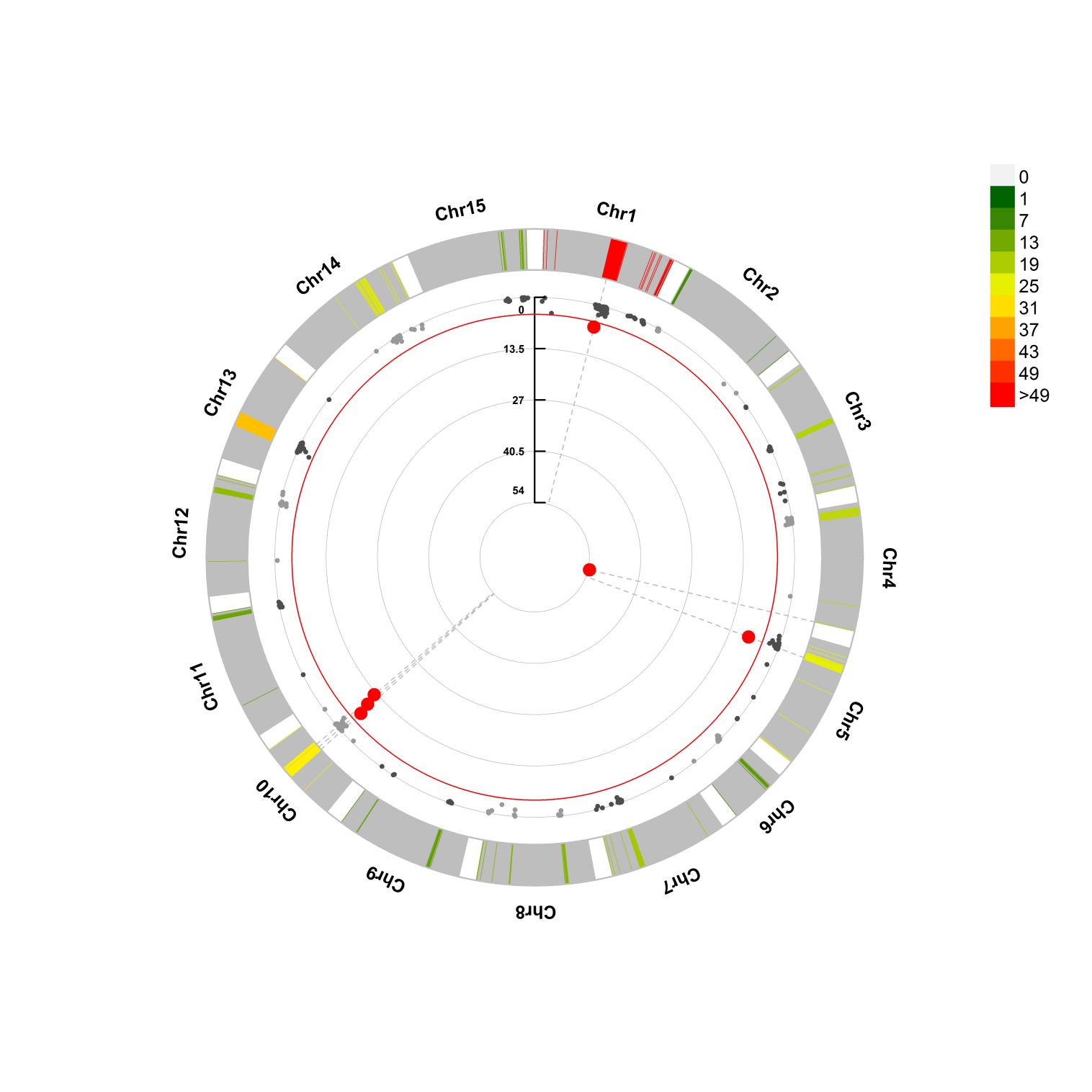


Figure S4 The Manhattan plot displaying on-target side effects of VPS29 in the IVW Phe-MR analysis


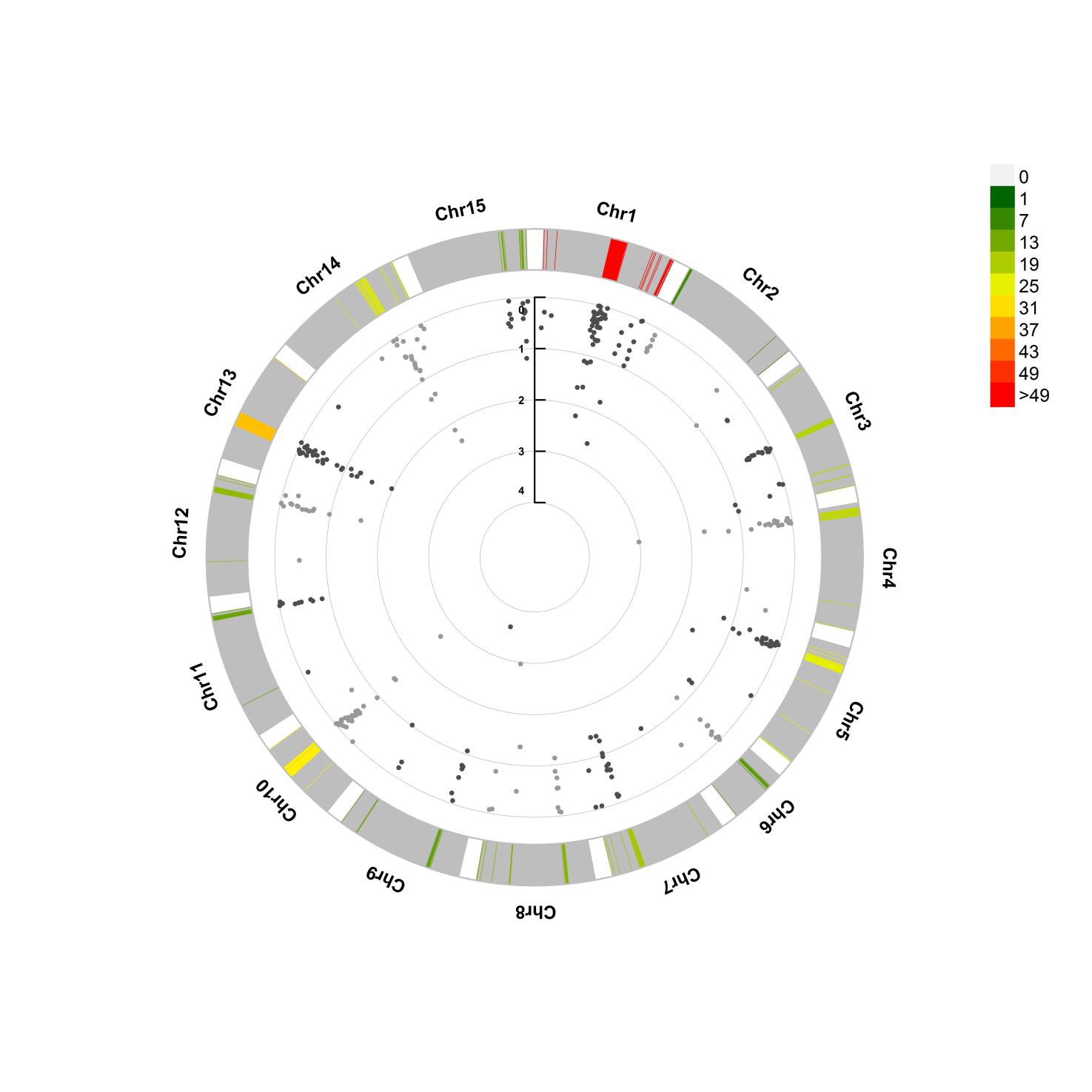


Figure S5 The Manhattan plot displaying on-target side effects of KIR2DS2 in the IVW Phe-MR analysis


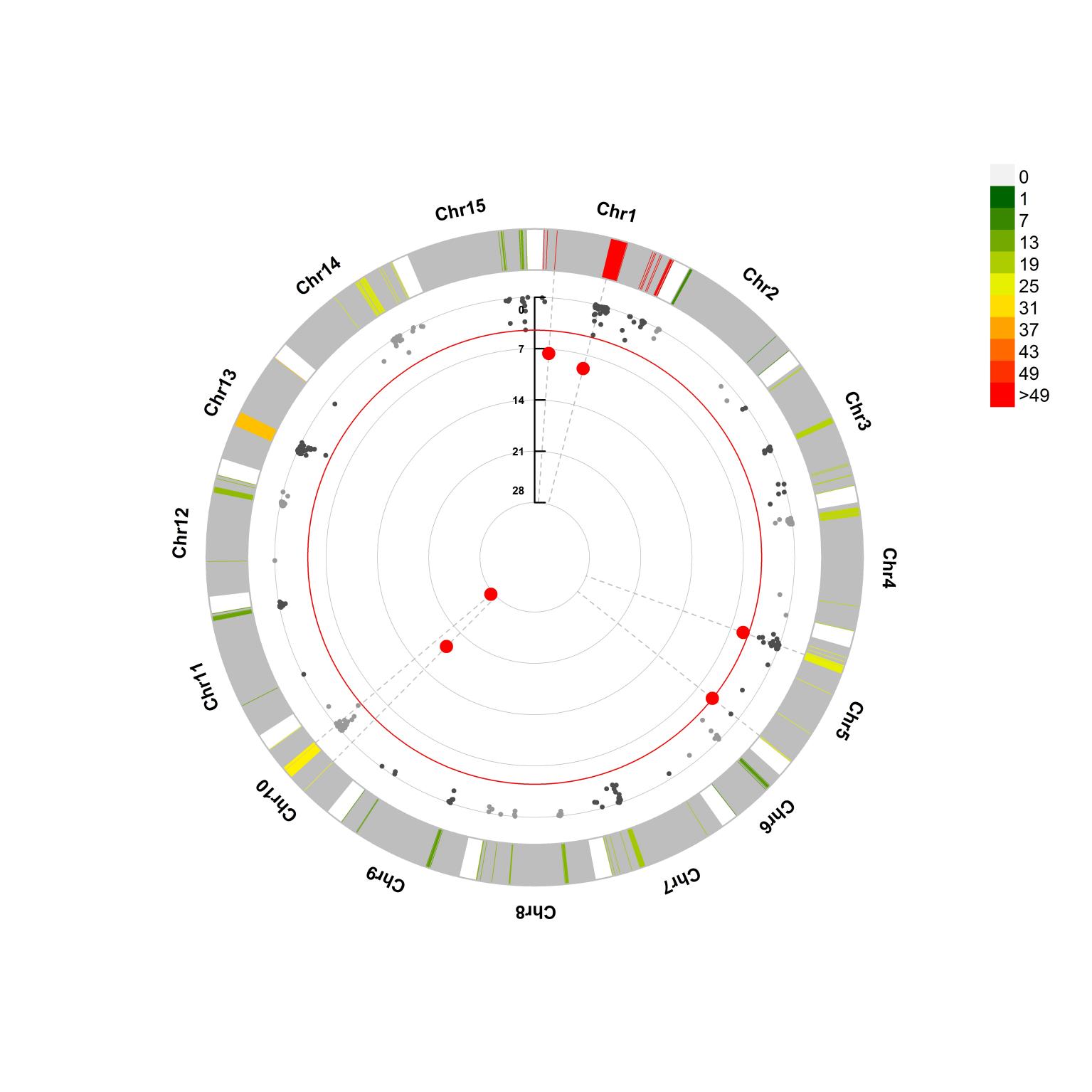


Figure S6 The Manhattan plot displaying on-target side effects of NAGAT in the IVW Phe-MR analysis
